# Diabetic mitochondria are resistant to palmitoyl CoA inhibition of respiration, which is detrimental during ischemia

**DOI:** 10.1096/fj.202100394R

**Published:** 2021-07-28

**Authors:** M. Kerr, K. M. J. H. Dennis, C. A. Carr, W. Fuller, G. Berridge, S. Rohling, C. L. Aitken, C. Lopez, R. Fischer, J. J. Miller, K. Clarke, D. J. Tyler, L. C. Heather

**Affiliations:** ^1^ Department of Physiology, Anatomy and Genetics University of Oxford Oxford UK; ^2^ Institute of Cardiovascular and Medical Sciences University of Glasgow Glasgow UK; ^3^ Target Discovery Institute University of Oxford Oxford UK; ^4^ Department of Physics University of Oxford Oxford UK; ^5^ Department of Clinical Medicine Aarhus University Aarhus Denmark; ^6^ Division of Cardiovascular Medicine Radcliffe Department of Medicine University of Oxford Oxford UK

**Keywords:** diabetes, energetics, fatty acids, heart, ischemia, mitochondria

## Abstract

The bioactive lipid intermediate palmitoyl CoA (PCoA) can inhibit mitochondrial ADP/ATP transport, though the physiological relevance of this regulation remains unclear. We questioned whether myocardial ischemia provides a pathological setting in which PCoA regulation of ADP/ATP transport would be beneficial, and secondly, whether the chronically elevated lipid content within the diabetic heart could make mitochondria less sensitive to the effects of PCoA. PCoA acutely decreased ADP‐stimulated state 3 respiration and increased the apparent *K*_m_ for ADP twofold. The half maximal inhibitory concentration (IC_50_) of PCoA in control mitochondria was 22 µM. This inhibitory effect of PCoA on respiration was blunted in diabetic mitochondria, with no significant difference in the *K*_m_ for ADP in the presence of PCoA, and an increase in the IC_50_ to 32 µM PCoA. The competitive inhibition by PCoA was localised to the phosphorylation apparatus, particularly the ADP/ATP carrier (AAC). During ischemia, the AAC imports ATP into the mitochondria, where it is hydrolysed by reversal of the ATP synthase, regenerating the membrane potential. Addition of PCoA dose‐dependently prevented this wasteful ATP hydrolysis for membrane repolarisation during ischemia, however, this beneficial effect was blunted in diabetic mitochondria. Finally, using ^31^P‐magnetic resonance spectroscopy we demonstrated that diabetic hearts lose ATP more rapidly during ischemia, with a threefold higher ATP decay rate compared with control hearts. In conclusion, PCoA plays a role in protecting mitochondrial energetics during ischemia, by preventing wasteful ATP hydrolysis. However, this beneficial effect is blunted in diabetes, contributing to the impaired energy metabolism seen during myocardial ischemia in the diabetic heart.

**Abbreviations**
AAC: ADP/ATP carrier
ANT: adenine nucleotide translocase
GPM: glutamate, pyruvate, malate
IFM: interfibrillar mitochondria
LCACoA: long chain acyl Co enzyme A
PCoA: palmitoyl CoA
PCarn: palmitoyl carnitine
SSM: subsarcolemmal mitochondria
T2D: type 2 diabetes

## 1 INTRODUCTION

In type 2 diabetes (T2D), cardiac fatty acid (FA) uptake, oxidation, and storage are all increased, resulting in increased dependence on FA for energy generation and increased accumulation of lipid intermediates within the cardiomyocyte.^1^ FAs are not only sources of energy for the heart, but lipid intermediates can also function as signalling molecules, regulating the transcription of FA‐target genes, directly regulating enzyme function, as well as driving post‐translational modification of proteins. Once FAs are imported into the cell they are activated by formation of a thioester bond to a CoA molecule, forming long chain acyl co‐enzyme As (LCACoA), including palmitoyl CoA (PCoA). Research has implicated PCoA as a biologically active signalling molecule,^2^, ^3^ and its concentration is increased in diabetes.^4^ A greater understanding of how increased PCoA may be influencing cell function is needed if we are to understand how diabetes negatively affects cardiac function. This will aid our understanding of how diabetes accelerates heart failure progression, given that cardiovascular disease is the leading cause of mortality in patients with diabetes.

Mitochondrial respiration accounts for 95% of ATP generation in the heart, and regeneration of ATP from ADP and phosphate (Pi) requires transport of these molecules across the highly impermeable mitochondrial inner membrane. Under normal physiological settings, ADP is imported into and ATP exported from the matrix by the ADP/ATP carrier (AAC), also known as the adenine nucleotide translocase (ANT). Whereas the phosphate carrier (PiC) has relatively weak control of the rate of oxidative phosphorylation, as shown by 60% depletion of PiC having no effect on mitochondrial respiration,^5^ the AAC is the most abundant protein in the inner mitochondrial membrane and has a high level of control over the rate of oxidative phosphorylation.^6^ The AAC is a monomeric α‐helical barrel, with a central cavity that can bind ADP or ATP, and can cycle between being open to the matrix or the intermembrane space.^7^

Research over many decades has shown that LCACoAs can reversibly inhibit the activity of the AAC.^8^, ^9^, ^10^ The potency of this inhibition has been postulated to be due to chemical and physical similarities between LCACoAs and the channel's substrates, ADP and ATP. However, the physiological relevance of this PCoA‐mediated regulation of AAC has been questioned, as in the healthy heart LCACoA concentrations are tightly controlled.^11^, ^12^ But in pathological states such as diabetes, LCACoA concentrations increase and may result in cellular conditions in which AAC activity and mitochondrial respiration are suppressed. Alternatively, the high lipid environment of diabetes may cause chronic adaptations within the cell, to allow mitochondrial function to continue regardless of the inhibitory presence of PCoA.

This study set out to investigate whether oxidative phosphorylation is acutely regulated by LCACoA in the heart, and to understand how this regulation changes in the lipotoxic environment of diabetes. Here we demonstrate that PCoA inhibits respiration in control mitochondria via regulation of the phosphorylation apparatus, but this effect is blunted in mitochondria from diabetic hearts. This has consequences during ischemia, as in the healthy heart PCoA serves to protect mitochondria by inhibiting the AAC, and preventing ATP hydrolysis via reverse ATP synthase activity. This protection of energy metabolism during ischemia is lost in diabetes, with diabetic hearts losing membrane potential and ATP more rapidly during ischemia.

## 2 METHODS

### 2.1 Rat model of T2D

All animal experiments conformed to Home Office Guidance on the Operation of the Animals (Scientific Procedures) Act, 1986 and were approved by a local ethics committee. T2D was induced as previously described,^13^ generating a mild model of the disease presenting with insulin resistance, mild hyperglycemia, hyperinsulinemia, and hyperlipidemia. Briefly, male Wistar rats (Envigo) were fed a high‐fat diet ad libitum (Special Diet Services, 829197, 60% calories from fat), and on day 14 they received a single low‐dose ip injection of streptozotocin (25 mg/kg body weight, w/w in citrate buffer, pH 4) following an overnight fast. Control rats were fed standard chow diet, and both groups of rats were maintained on their respective diets for a further 6 weeks.

### 2.2 Mitochondrial isolation and respiration

Subsarcolemmal (SSM) and interfibrillar (IFM) mitochondria were isolated from the heart according to our previously published protocol.^14^ Mitochondria were respired in a Clark‐type oxygen electrode (Strathkelvin Instruments) as previously described,^14^ using glutamate (20 mM), pyruvate (10 mM) and malate (5 mM) (GPM) as substrates, with state 3 respiration induced by addition of 200 µM of ADP. Mitochondria were incubated with GPM and either PCoA (10 µM), PCoA (10 µM) with carnitine (Carn 5 mM) or palmitoyl carnitine (PCarn 5 µM) for 3 minutes, followed by addition of ADP (200 µM) or FCCP (5 µM), to induce state 3 or uncoupled respiration, respectively.

For the generation of *K*_m_ and *V*_max_ values, mitochondrial respiration in the presence of PCoA or PCoA + Carn were carried out as described above (following a 3‐minutes incubation), with the exception that the concentration of ADP was varied between 40 and 1000 µM, prior to the fitting of data to Michaelis‐Menton kinetics via nonlinear regression. For the measurement of inhibitory concentration (IC_50_) values, experiments were performed as described above with 200 µM ADP to induce state 3 respiration, but with the concentration of PCoA varied between 0 and 52 µM. All derived quantities were calculated using nonlinear regression analysis in GraphPad Prism 8.

### 2.3 Gene expression

RNA was extracted from frozen cardiac tissue using the Qiagen RNeasy Mini Kit, and converted into cDNA using a high‐capacity cDNA reverse transcriptase kit (Applied Biosystems). Quantitative polymerase chain reaction was performed using the SYBR detection method, and a StepOnePlus Real‐Time PCR system (Applied Biosystems). Primers for AAC1 (sense AGCAGTTCTGGCGCTACTTC, antisense CCATTGAACTCACGCTGGGA), AAC2 (sense CAAAGGGAATGCTCCCGGAT, antisense ATCTTCCGCCAGCAGTCAAG) and GAPDH (sense AGTATGTCGTGGAGTCTACTGGTG, antisense TGAGTTGTCATATTTCTCGTGGTT) were used. Analysis was carried out using the 2^−ΔΔCT^ method.

### 2.4 Western blotting

Lysates prepared from frozen cardiac tissue and isolated mitochondria were used for western blotting as previously described.^15^ Antibodies to AAC1 and AAC2 were custom generated by Eurogentec, raised to residues 144‐157, which displayed significant divergence between isoforms. Primary antibodies to total AAC (Santa Cruz sc9299) and the PiC (Abcam ab67121) were also used. Cyclophilin B (Abcam ab178397) and glutamate dehydrogenase (Abcam ab166618) were used as housekeeper proteins for cardiac and isolated mitochondrial westerns, respectively. Bands were quantified using LI‐COR C‐DiGit technology, and data expressed relative to control.

### 2.5 Molecular analyses

Citrate synthase activity was measured according to the protocol of Srere.^16^ ATP synthase activity was measured using a kit from Abcam (ab109714‐96). Palmitoylation was assessed using a Badrilla CAPTUREome S‐Palmitoylated Protein Kit to isolate the palmitoylated proteins from SSM and IFM populations, followed by western blotting for AAC1 and AAC2. Caveolin 3 was used as a positive control for palmitoylation, due to its high degree of palmitoylation (BD Biosciences 610420).

Acetylation of AAC in SSM and IFM was assessed using mass spectrometry, following immunoprecipitation using an anti‐acetyl lysine antibody (Cell Signalling Technology, 9441S and 9681S) to isolate acetylated proteins. The immobilised proteins were digested by adding SMART trypsin (Thermo Fisher), and the eluted peptides were desalted using SepPak reverse phase columns and evaporated to dryness. Mass Spectrometry data was acquired by an Orbitrap Fusion Lumos after chromatographic separation with a Dionex Ultimate 3000 (Thermo Fisher) according to our previously published protocol.^17^ Peptides were separated on an Easyspray column (ES803) with a gradient of 5%‐35% acetonitrile in 0.1% formic acid/5% DMSO over 60 minutes. For label‐free quantitation, data was analyzed with Progenesis QI and searched against rattus norvegicus database with PEAKS 8.5. Quantitative data were then further analyzed using Perseus.^18^ The mass spectrometry proteomics data have been deposited to the ProteomeXchange Consortium via the PRIDE partner repository with the dataset identifier PXD024500.

Long chain acyl CoAs were extracted from cardiac tissue according to the method of Hoppel et al.^19^ Samples were evaporated to dryness, then resuspended in 60% acetonitrile with 0.1% formic acid. Samples were injected on to the C17 BEH 1.7 µm column, and separated with a 35‐minutes gradient from 2% acetonitrile with 0.1% formic acid to 100% acetonitrile with 0.1% formic acid. Data was acquired with an Agilent 6560 IM Q‐TOF, target ions were extracted using Mass Hunter Qualitative Analysis software, and related to standard curves of the predominant LCACoAs.

Using the values obtained, the mitochondrial LCACoA was calculated using the equation below:

$$\text{Matrix LCACoA}=\frac{\left(\text{Specific gravity of cardiac tissue}\right)\times \left(\text{Myocardial concentration of LCACoA}\right)}{\left(\text{Mitochondrial cell fraction}\right)\times \left(\text{Fraction of LCACoA localised to the matrix}\right)}.$$

With the values taken from the literature for the specific gravity of cardiac tissue,^20^ mitochondrial cell fraction^21^ and % of LCACoA in the matrix.^22^

### 2.6 Mitochondrial model of ischemia

Respiration media was supplemented with 5‐µM safranine O in 96‐well black plates, and membrane potential was measured using a FLUOstar Omega plate reader using an excitation wavelength of 495 nm and emission wavelength of 586 nm. Absolute values were calculated using a potassium clamp as described by Akerman and Wikstrom,^23^ with valinomycin (40 ng/mL) added prior to addition of increasing concentrations of KCl. Membrane potential was calculated using the Nernst equation defined by:

$$\Delta \Psi =60\times \log\frac{K_{\text{in}}^{+}}{K_{\text{out}}^{+}}$$

Substrates glutamate (20 mM), malate (5 mM) and pyruvate (10 mM), ADP (200 µM), ATP (10 mM), and inhibitors myxothiazol (8 µM), carboxyatractyloside (CATR 2.5 mM), oligomycin (10 µM), and BMS199264 (3 µM) were added, and fluorescence measured after stabilisation.

### 2.7 ^31^P‐magnetic resonance spectroscopy of isolated perfused heart

Rat hearts were excised and arrested in ice‐cold Krebs Henseleit (KH) buffer, the aorta was cannulated, and the hearts were perfused in Langendorff‐mode with warmed KH buffer according to our previous publication.^24^ A balloon containing phenylphosphoric acid (PPA) was inserted into the left ventricle to allow absolute quantification of energy metabolites ATP and phosphocreatine (PCr).^24^ The contracting heart was placed in a glass NMR sample tube and lowered into the isocentre of an 11.7 T vertical bore preclinical MRI scanner, with temperature controlled by forced air at 37°C. Localiser images were obtained to check the location of the heart prior to shimming and the acquisition of a fully‐relaxed ^31^P‐MRS spectra (15 s TR, 40 averages, 90° flip angle, 10 kHz bandwidth, 2048 complex points). This was followed by the acquisition of time‐resolved spectra (1 s TR, 60 averages, ~50° flip angle, 10 kHz bandwidth, 2048 complex points) throughout 15 minutes of full flow perfusion, followed by 32 minutes of low‐flow ischemia at 0.5 mL/minutes/gww. After the end of this acquisition, a further 50 µL of PPA was injected into the left ventricular balloon prior to the acquisition of another fully‐relaxed spectrum, with this process repeated to allow for absolute quantification of metabolite levels.

After acquisition, spectra were quantified using a MATLAB implementation of the AMARES algorithm using previously published prior knowledge that takes into account the known spectral locations of ATP and its J‐coupling, PCr, PPA, and Pi, which permits the absolute quantification of intracellular energy metabolite concentrations.^25^, ^26^, ^27^ Quantification of the ischaemic time course was analyzed by summing spectra in 4‐minutes groups, then fitting the ATP and PCr amplitudes to a simple mono‐exponential decay curve.

### 2.8 Statistics

**FIGURE 1:**
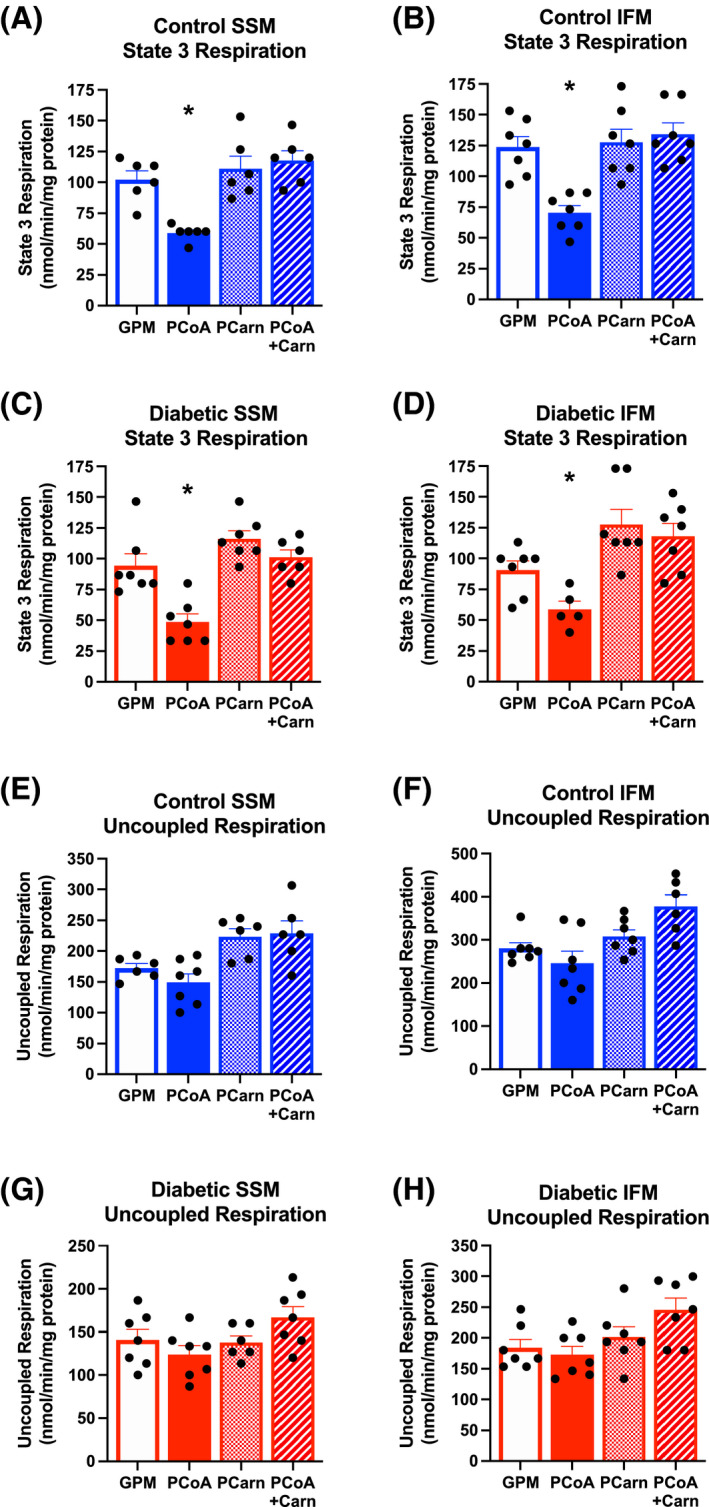
Palmitoyl CoA acutely decreases state 3 respiration. State 3 respiration rates in control (A,B) and diabetic (C,D) SSM and IFM when respiring on glutamate, pyruvate and malate (GPM), plus palmitoyl CoA (PCoA), palmitoyl carnitine (PCarn), or palmitoyl CoA with carnitine (PCoA + Carn). FCCP‐uncoupled respiration rates in control (E,F) and diabetic (G,H) SSM and IFM when respiring on GPM, plus PCoA, PCarn, or PCoA + Carn. *P < .05 versus GPM alone

**FIGURE 2:**
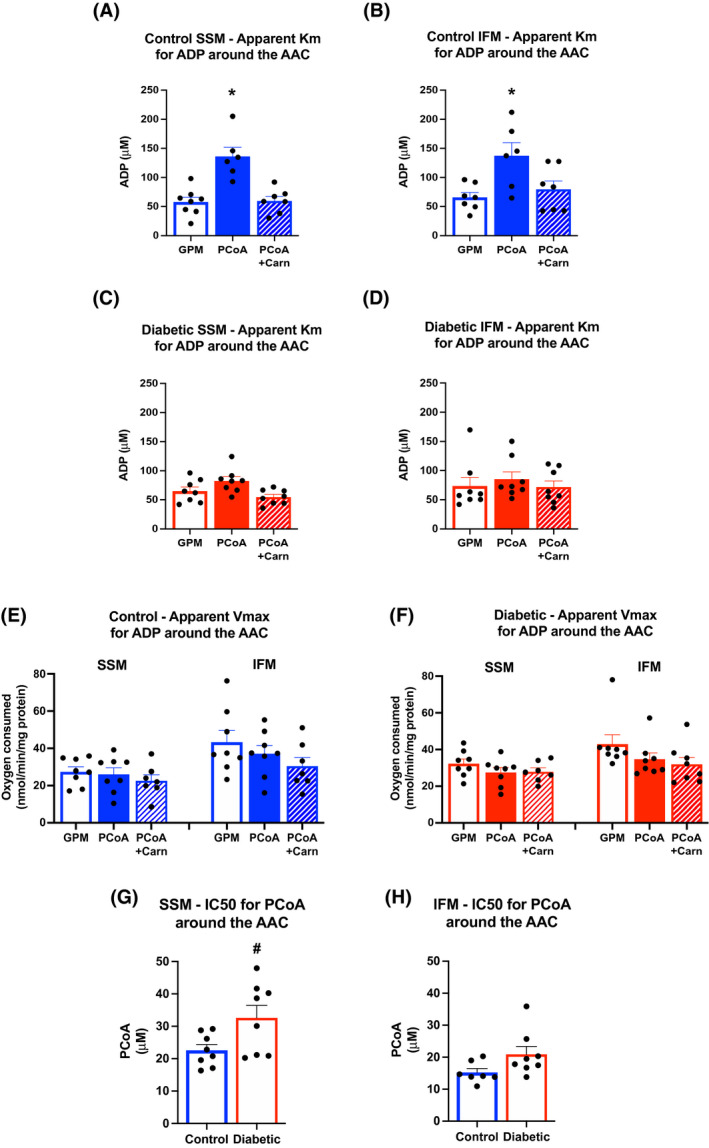
Diabetic mitochondria have altered kinetics for palmitoyl CoA inhibition. Michaelis‐Menton kinetic‐like curves were generated to calculate *K*_m_ (A–D) and *V*_max_ (E–F) around the AAC in control (A,B) and diabetic (C,D) SSM and IFM when respiring on glutamate, pyruvate and malate (GPM), plus palmitoyl CoA (PCoA) or palmitoyl CoA with carnitine (PCoA + Carn). IC_50_ for palmitoyl CoA around the AAC in control and diabetic SSM (G) and IFM (H). *P < .05 versus GPM alone, ^#^ *P* < .05 versus control

**FIGURE 3:**
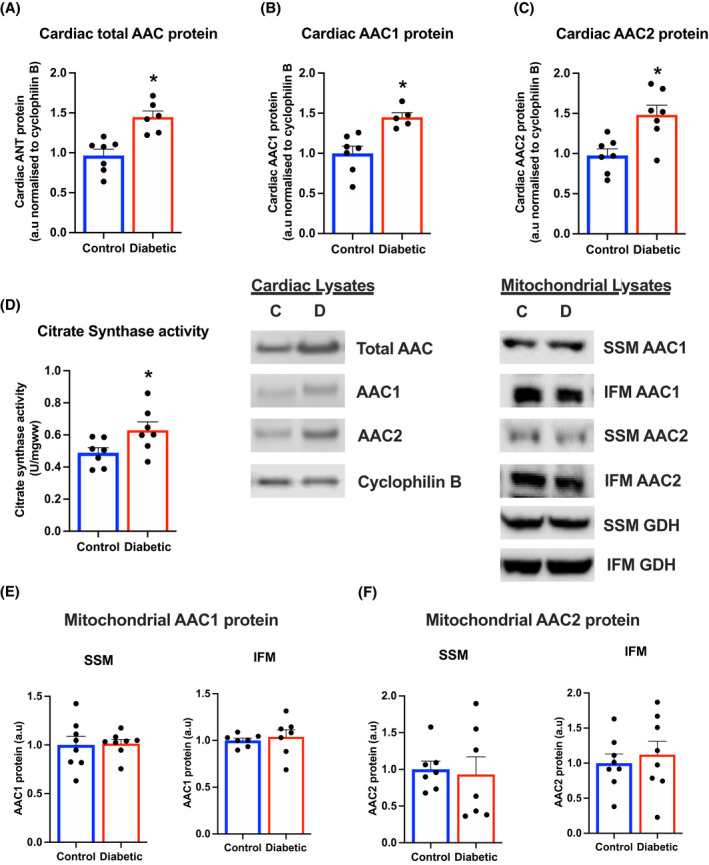
No change in mitochondrial AAC isoform in diabetes. Whole heart expression of total AAC (A), AAC1 (B), and AAC2 (C) in control and diabetic hearts. Citrate synthase activity (D) in cardiac homogenates, as an indicator of mitochondrial number, from control and diabetic hearts. Mitochondrial AAC1 (E) and AAC2 (F) protein in SSM and IFM, isolated from control and diabetic hearts. Cyclophilin B and glutamate dehydrogenase (GDH) were the housekeeper proteins for the cardiac and mitochondrial lysates, respectively. *P < .05 versus control

**FIGURE 4:**
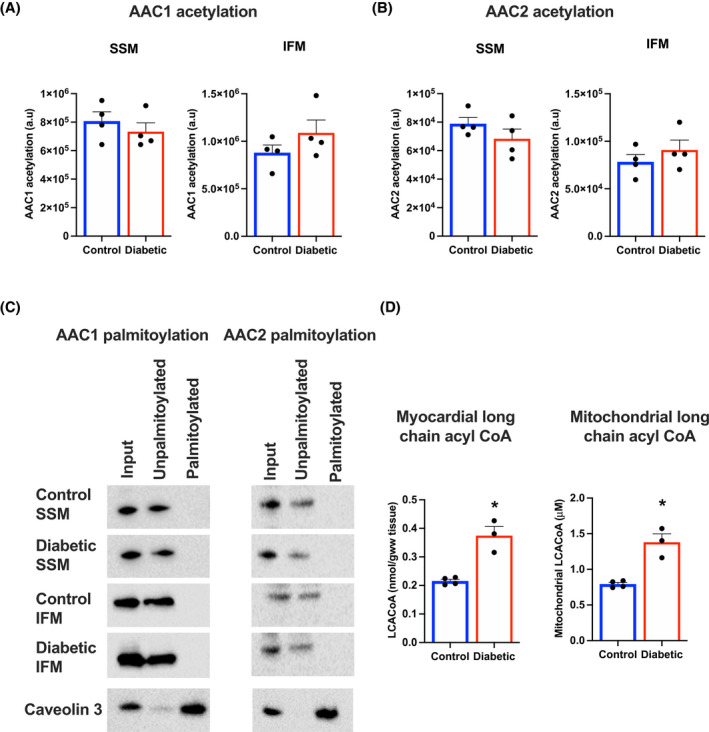
No change in AAC posttranslational modifications, despite increased myocardial long chain acyl CoA in diabetes. AAC1 (A) and AAC2 (B) acetylation in SSM and IFM, isolated from control and diabetic hearts. Representative blots demonstrating no evidence of AAC1 or AAC2 palmitoylation in SSM and IFM isolated from control and diabetic hearts (C). Caveolin 3 is used as a positive control to demonstrate palmitoylation of this protein in the mitochondrial extracts. Myocardial long chain acyl CoA concentrations (D) from control and diabetic hearts, and the respective mitochondrial LCACoA concentrations. *P < .05 versus control

**FIGURE 5:**
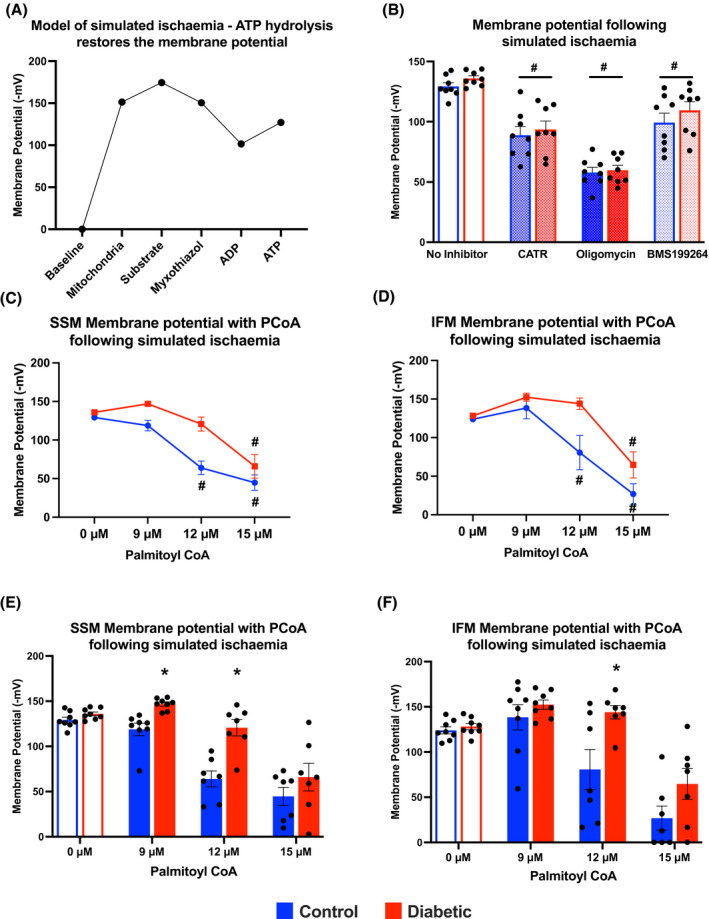
Palmitoyl CoA prevents membrane repolarisation during ischemia, but this effect is blunted in diabetes. Ischemia was modelled in isolated mitochondria using myxothiazol to block electron transport chain activity and ADP to deplete the proton gradient (A). Addition of ATP under these simulated ischaemic conditions increased the membrane potential, by ATP hydrolysis via reverse ATP synthase activity. Membrane potential in SSM (B) from control and diabetic hearts, when the AAC inhibitor carboxyatractyloside (CATR), the ATP synthase inhibitor oligomycin and the inhibitor of the reverse hydrolase activity of ATP synthase, BMS199264, were added prior to ATP. Membrane potential in SSM (C and E) and IFM (D and F) from control and diabetic hearts when palmitoyl CoA was added prior to ATP. ^#^ *P* < .05 versus no inhibitor/0 µM palmitoyl CoA, *P < .05 versus control

**FIGURE 6:**
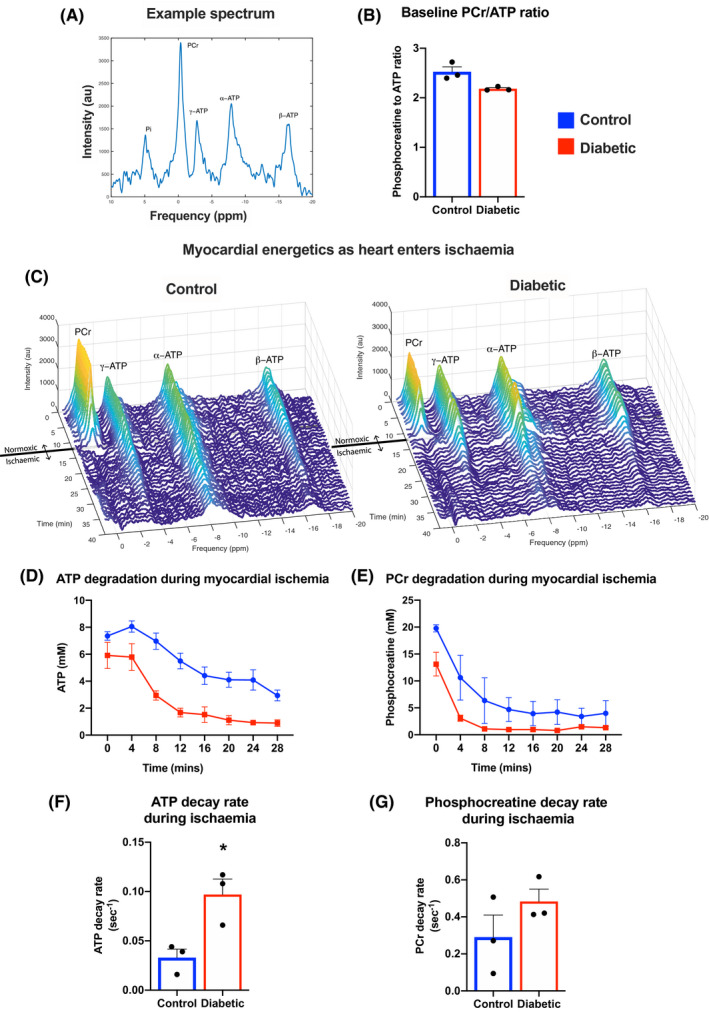
Diabetic hearts lose ATP more rapidly than control hearts during ischemia. Myocardial energetics were measured in perfused contracting hearts using ^31^P‐magnetic resonance spectroscopy, with an example spectrum in panel A. Baseline phosphocreatine (PCr) to ATP ratios in control and diabetic hearts (B). Example waterfall plots of control and diabetic (C) hearts prior to and during ischemia. ATP and PCr concentrations (D,E) and the calculated decay rates (F,G) during ischemia in control and diabetic hearts. *P < .05 versus control

Data are presented as mean ± SEM, and considered significant at a *P* value of less than .05, using GraphPad Prism 8. Data were analyzed using one‐way ANOVA with Dunnett's test for multiple comparison (Figures 1 and 2A‐F). A two‐way ANOVA was used for analysis of Figure 5, with Dunnett's test for multiple comparison (panels B‐D) or Sidak's multiple comparison test (Figure 6E,F). When two groups were compared, an unpaired *t* test was used (Figures 2G,H, 3, 4, and 6).

## 3 RESULTS

### 3.1 PCoA acutely inhibits state 3 respiration

Subsarcolemmal and IFM cardiac mitochondria were respired under state 3 ADP‐stimulated conditions with glutamate, pyruvate and malate (GPM) as substrates. Incubation of the mitochondria for 3 minutes with this GPM mix supplemented with PCoA, resulted in decreased state 3 respiration in SSM and IFM from control and diabetic hearts (Figure 1A‐D). For PCoA to be oxidised, it must first be imported into the mitochondrial matrix following conjugation with carnitine. In contrast to PCoA, the inhibition of respiration did not take place when palmitoyl carnitine (PCarn) was added, demonstrating that PCoA inhibition was independent of its oxidation. Similarly, addition of carnitine to PCoA (PCoA + Carn) had no effect on respiration, thus, removing the CoA moiety of the LCACoA form also removed the inhibition. When experiments were repeated using the uncoupler FCCP, which bypasses the phosphorylation apparatus to stimulate respiration, the inhibitory effect of PCoA was no longer present (Figure 1E‐H). This demonstrates that the site of PCoA inhibition was within the phosphorylation apparatus.

### 3.2 Diabetic mitochondria have altered kinetics for PCoA inhibition

The kinetics of PCoA inhibition were probed through the generation of Michaelis‐Menten‐like kinetic curves, whereby the concentration of ADP was varied, and state 3 respiration was measured. In the presence of GPM substrate alone, both control and diabetic mitochondria had similar *K*_m_ measurements (Figure 2A‐D). When PCoA was added to GPM, the apparent *K*_m_ increased by 235% and 208% in SSM and IFM, respectively, from control hearts (Figure 2A,B). This increase in *K*_m_ with PCoA occurred independently of any change in the *V*_max_ (Figure 2E), demonstrating that the mechanism of PCoA inhibition was competitive. When PCoA was added in the presence of carnitine, to allow for its oxidation in the matrix, no change in *K*_m_ was found when compared with GPM alone (Figure 2A,B).

In contrast to the findings in control mitochondria, diabetic mitochondria displayed no significant change in *K*_m_ in the presence of PCoA in either SSM or IFM, compared with GPM alone (Figure 2C,D,F). No difference in *K*_m_ between GPM, PCoA and PCoA with carnitine was found in diabetic mitochondria. Thus, the competitive inhibition of PCoA for ADP‐stimulated respiration at the phosphorylation apparatus was blunted in mitochondria from diabetic rats.

In order to investigate this change in PCoA kinetics further, ADP‐stimulated respiration rates were measured after incubation with a range of PCoA concentrations, to determine the IC_50_ for PCoA inhibition (Figure 2G,H). IC_50_ values demonstrated that while both control and diabetic mitochondria could be inhibited by PCoA, diabetic SSM required 44% more PCoA than controls to induce the same inhibition. IFM had a significantly lower IC_50_ than SSM, and diabetic IFM had a 37% increase compared with control IFM, though this failed to reach significance (*P* = .06).

### 3.3 Mitochondrial AAC protein isoform expression and post‐translational modifications are unchanged by diabetes

To try to understand the changes in sensitivity to PCoA inhibition in diabetes, we looked at expression of the phosphorylation apparatus. Given the high control coefficient of the AAC^6^ and the order of magnitude higher reported sensitivity of the AAC to lipid intermediates, it is most likely to be the site of regulation. However, we first ruled out changes in ATP synthase activity and PiC protein levels between control and diabetic mitochondria (Figure S1).

The different AAC isoforms have different kinetics for ADP transport, which may also result in different sensitivities to LCACoA inhibition. The mRNA expression of AAC1 was significantly increased in diabetic hearts compared with control hearts, whereas the mRNA expression of AAC2 was not significantly different between groups (Figure S1). To see if this resulted in a change in cardiac protein levels of the AAC, we measured total cardiac AAC protein using an antibody that would bind to both isoforms. This demonstrated that hearts from diabetic rats had a 49% increase in cardiac AAC protein levels (Figure 3A). We generated our own antibodies to the individual AAC isoforms, raised to regions that displayed significant divergence between isoforms. This revealed both AAC1 and AAC2 protein were increased in diabetic hearts by 45% and 51%, respectively (Figure 3B,C). Thus, at first sight it appeared that changes in AAC protein expression may play a role in mitochondrial dysfunction in diabetes. However, we also identified that citrate synthase activity, a marker of mitochondrial content, was also increased in diabetic hearts (Figure 3D), which raised the question whether increased cardiac AAC protein levels were simply due to increased mitochondrial abundance in diabetic hearts. Therefore, we measured AAC isoform protein levels in the two mitochondrial populations isolated from the heart. There were no differences in AAC1 or AAC2 protein levels in SSM or IFM from diabetic hearts compared with controls, demonstrating that increases in whole heart AAC protein were solely due to increases in mitochondrial abundance in diabetes (Figure 3E,F). Thus, changes in sensitivity to PCoA were not due to differential expression of AAC isoforms in diabetes.

Given that no difference in isoform protein expression could be identified, specific post‐translational modifications, acetylation and palmitoylation, were measured, which have been mechanistically implicated in high fat exposure and nutrient excess. There were no significant differences in AAC1 or AAC2 acetylation between control and diabetic SSM or IFM (Figure 4A,B). In addition, we found no evidence that AAC1 or 2 were post‐translationally palmitoylated (Figure 4C). In control and diabetic mitochondria, AAC isoforms were recovered in the unpalmitoylated fraction, with no evidence of either AAC protein in the palmitoylated fraction. This was in contrast to caveolin 3, a known palmitoylated protein that localises to mitochondria, which was recovered in the palmitoylated fraction from isolated mitochondria.

The concentration of the long chain acyl CoA's was measured within the myocardium, and was found to be increased by 74% in diabetic hearts (Figure 4D). Due to the known distribution of LCACoAs within the cardiomyocyte, the concentration of LCACoA's within the mitochondria could be calculated, and was found to be within the µM range, at the lower end of the range calculated for the IC_50_ values. Liepinsh et al showed in response to ischemia cardiac LCACoA concentrations increased to 11 nmol/gww,^28^ which we calculate would give a mitochondrial concentration of 40 µM, which is within the range of our IC_50_ values. Thus, the inhibitory effects of LCACoA inhibition on the AAC in diabetes may not be physiologically relevant under baseline conditions, but instead become significant in response to pathological ischemia.

### 3.4 PCoA protects against membrane repolarisation during ischemia but is blunted in diabetes

To test our hypothesis that PCoA inhibition of the AAC may serve a role in ischemia, a model of mitochondrial ischemia was developed. Mitochondrial membrane potential was measured in the presence of substrate and was found to be between −167 and −174 mV across the different mitochondrial populations (Figure 5A), in agreement with other published values.^29^ Addition of myxothiazol, to block complex III, and ADP, decreased the membrane potential and this was not regenerated, confirming electron movement along the chain was blocked, akin to ischemia. It has been estimated that during ischemia, a proportion of ATP is wasted due to hydrolysis by reverse ATP synthase.^30^ In our model of mitochondrial ischemia, addition of ATP resulted in membrane potential regeneration. This membrane repolarisation was most likely via ATP import through the AAC, ATP hydrolysis by reversal of the ATP synthase and reverse proton pumping. To confirm this mechanism of membrane repolarisation, CATR an inhibitor of the AAC, oligomycin an inhibitor of ATP synthase, and BMS199264 an inhibitor of the reverse hydrolase activity of ATP synthase were used (Figure 5B). All three compounds prevented ATP repolarising the membrane in SSM, confirming that membrane repolarisation during ischemia was via ATP hydrolysis by the phosphorylation apparatus.

In both SSM and IFM populations, PCoA was also able to prevent this membrane repolarisation by ATP, in a concentration‐dependent manner, with significance present from 12 µM onwards in control mitochondria, but only at 15 µM in diabetic mitochondria (Figure 5C,D). When a direct comparison was made between groups, there was a significant difference in the ability to prevent membrane repolarisation between control and diabetic mitochondria at 9 and 12 µM in SSM, and at 12 µM in IFM (Figure 5E,F). Thus, in control hearts during ischemia, LCACoA prevented membrane repolarisation via wasteful ATP hydrolysis, but this effect was blunted in diabetic mitochondria.

### 3.5 Diabetic hearts lose ATP more rapidly than control hearts during ischemia

Intact contracting control and diabetic hearts were perfused within an 11.7 T magnet, and ^31^P‐spectroscopy was used to measure high energy phosphates ATP and PCr, within the myocardium (Figure 6A). Under baseline pre‐ischaemic conditions myocardial energetic status, as assessed by the PCr to ATP ratio, was not significantly different between control and diabetic hearts, in agreement with our previously published findings in this early‐stage model of T2D^24^ (Figure 6B). Hearts were then subjected to low‐flow ischemia, and the rates of ATP and PCr degradation measured (Figure 6C‐E). The ATP decay rates were 3‐fold higher in diabetic hearts during ischemia than in control hearts (Figure 6F). This occurred without any significant difference in the PCr decay rates between the groups (Figure 6G). Whilst differences in whole‐heart ATP concentrations are a combination of changes in ATP synthesis balanced with ATP degradation, this data supports our mitochondrial findings that during ischemia diabetes causes an accelerated loss of cellular ATP.

## 4 DISCUSSION

Lipid intermediates accumulate within the heart in diabetes, and understanding their role in regulating cellular processes will help us understand why cardiomyocyte function is compromised in diabetes. Here we demonstrate that PCoA can acutely inhibit mitochondrial respiration in the heart, via inhibition of the phosphorylation apparatus. We demonstrate that this inhibition is blunted in the diabetic heart, an environment with elevated concentrations of LCACoA's. While this regulation may not be sufficient to inhibit respiration under normal physiological concentrations, we demonstrate a beneficial role for PCoA‐mediated AAC inhibition during ischemia in control hearts. Thus, lipid intermediates can play a role in protecting the heart in ischemia, but this is blunted and protection is decreased in diabetes during myocardial ischemia.

### 4.1 Diabetic mitochondria are resistant to the suppressive effects of PCoA on respiration

Control mitochondria acutely decrease mitochondrial respiration when exposed to PCoA, an effect that is exclusive to the CoA ester form of palmitate, and is absent if the PCoA can be transported into the mitochondria for oxidation. The site of PCoA‐mediated respiratory inhibition can be localised to the phosphorylation apparatus, and both experimental and computational‐based modular kinetic analysis have confirmed the predominant site of PCoA control is the AAC.^8^, ^9^, ^10^, ^31^ This is due to the orders‐of‐magnitude greater sensitivity of the AAC than the other carriers, with the AAC 250‐fold more sensitive to PCoA than the PiC,^32^ and to the high level of control exerted by the AAC over the phosphorylation apparatus, which is estimated to be between 62%‐75% under physiological conditions,^6^ particularly in the presence of PCoA.^10^, ^31^ Mitochondria isolated from T2D hearts are significantly less sensitive to PCoA inhibition, with an elevated IC_50_ and no significant increase in the *K*_m_ for ADP when PCoA is present. This insensitivity to the effects of PCoA may represent an adaptation to the high fat environment of the T2D heart, given that diabetic mitochondria will be chronically bathed in higher concentrations of lipid intermediates.

Suppression of mitochondrial respiration via competitive inhibition of the AAC by LCACoA's has been reported previously in liver and skeletal muscle. Here we show this is a preserved mechanism also occurring in the heart, and is an interesting observation given that the heart is a primary consumer of FAs and has higher ATP demand per gram of tissue than any other organ. At first glance, it would appear undesirable for the heart to have its energy generation compromised by an intermediate of its predominant energy generating fuel, which led us to consider situations when it may be beneficial for this regulation to occur.

### 4.2 PCoA protects mitochondrial energetics during ischemia, but this effect is blunted in diabetes

It has been debated for many years as to whether there is a physiological role for regulation by PCoA of the AAC. Based on our measured IC_50_ values and LCACoA concentrations, we would suggest any control under normal conditions would be minimal. However, our data provides an alternative role for this regulation in the pathological setting of ischemia, when the concentrations of LCACoA have been demonstrated to increase by up to 300%.^33^ During ischemia, loss of the membrane potential due to the absence of oxygen removes the drive for ATP synthase to function in the forward direction, and allows for reverse ATP hydrolysis. Therefore, ATP synthase generates, rather than uses, the proton gradient in ischemia. Identification of the mitochondrially‐located Inhibitory Factor 1 demonstrated that cells had evolved a mechanism to try to limit this wasteful ATP hydrolysis, by inhibiting ATP synthase operating in reverse.^34^, ^35^, ^36^ Given that there is an endogenous system in place to prevent reverse ATP synthase activity it would not be unreasonable to wonder if there was an endogenous system in place to prevent reverse AAC activity. Our work shows that PCoA can prevent ATP import into the mitochondria by the AAC during ischemia, thereby preventing wasteful ATP hydrolysis. This mechanism would be beneficial for the heart under ischaemic conditions, when ATP becomes an even scarcer resource, only being generated by the low yielding glycolytic pathway.

In diabetes, we have shown that the ability of PCoA to preserve the membrane potential is suppressed. This would result in diabetic hearts losing ATP at a greater rate during ischemia, and becoming energy depleted more rapidly. To extrapolate our findings from isolated mitochondria to the intact beating heart, we demonstrate that as the diabetic heart enters ischemia the rate of ATP loss is greater than that seen in control hearts, under the same ischaemic flow rate. The diabetic heart suffers a “double whammy” under these ischaemic conditions, as not only is ATP hydrolysis by the mitochondria accelerated but the upregulation of anaerobic glycolysis and cytosolic ATP synthesis is suppressed.^13^ This dysregulation by lipids in diabetes may contribute to the poorer prognosis following myocardial infarction in diabetic patients.^37^

### 4.3 Mechanistic changes in AAC do not currently explain changes in PCoA sensitivity in diabetes

Genetic studies on AAC have generated confusing findings, with both overexpression and deletion resulting in improved insulin sensitivity.^38^, ^39^ Increased AAC expression and protein levels were identified in the diabetic heart, however, upon further investigation it became apparent that this was due to increased mitochondrial abundance and mitochondrial biogenesis within the heart in diabetes.^40^, ^41^ The two AAC isoforms have different kinetic properties,^42^, ^43^ but at the level of the individual mitochondria we found no evidence of changes in AAC protein concentration or the ratio of AAC isoforms. While multiple post‐translational modifications have been suggested for the AAC, a recent review by Kunji and Ruprecht^44^ has questioned if most of these are artifacts of sample preparation. AAC1 has been shown to be acetylated, and computational modelling has demonstrated that increased AAC1 acetylation will decrease the affinity of the transporter for its substrate.^45^ However, AAC acetylation was not changed by diabetes, in agreement with our previous publication.^24^ Similarly, we could find no evidence that AAC was palmitoylated in either control or diabetic hearts, despite the substrate for palmitoylation being PCoA. Thus, we can conclude that the change in sensitivity to PCoA in diabetes was not mediated by transcriptional/translational control of the AAC, which leads us to postulate if there could be an acute modification during the 3 minutes of PCoA exposure (occurring exclusively in the mitochondria without involvement of other cellular compartments), though this is purely speculative.

Understanding the effects of lipid intermediates on mitochondrial and cellular functions is important in lipid‐overloaded settings such as diabetes and ischemia, to understand the pathophysiological mechanisms of these diseases. Previous research has shown that long chain acyl carnitines, such as PCarn can exert deleterious effects on calcium handling, membrane permeability and respiratory coupling in cardiac mitochondria.^46^, ^47^, ^48^, ^49^ Our current study demonstrates that the inhibitory effects of PCoA can be prevented by conjugating to carnitine, thus, the PCarn is protective in this setting. The class of lipid intermediates is vast, and understanding which intermediates are particularly toxic to the cell in each specific pathophysiological settings will be important from a therapeutic targeting perspective.

In conclusion, LCACoA can inhibit the phosphorylation apparatus and decrease respiration via interaction with AAC, and while this is unlikely to play a significant role in regulating energetics under normal physiological conditions, in ischemia this prevents wasteful ATP hydrolysis to regenerate the membrane potential. In T2D, this regulation of the AAC is blunted and mitochondria become resistant to the suppressive effects of PCoA, resulting in more rapid ATP depletion during ischemia.

## CONFLICT OF INTEREST

The authors have stated explicitly that there are no conflicts of interest in connection with this article.

## AUTHOR CONTRIBUTIONS

M. Kerr, W. Fuller, K. Clarke and L.C. Heather designed research. M. Kerr, K.M.J.H. Dennis, C.A. Carr, G. Berridge, S. Rohling, C.L. Aitken, and C. Lopez performed research. M. Kerr, D.J. Tyler, J.J. Miller and L.C. Heather analyzed data. M. Kerr and L.C. Heather wrote the manuscript.

## Supporting information

Fig S1
