# Supplementary material for: Diabetic mitochondria are resistant to palmitoyl CoA inhibition of respiration, which is detrimental during ischemia
Source: FASEB J. 2021 Jul 28;35(8):e21765. doi: 10.1096/fj.202100394R (PMC8662312; doi:10.1096/fj.202100394R)
Supplement: Supplementary file 1 — Fig S1 [file FSB2-35-e21765-s001.pdf]

Supplementary Figure

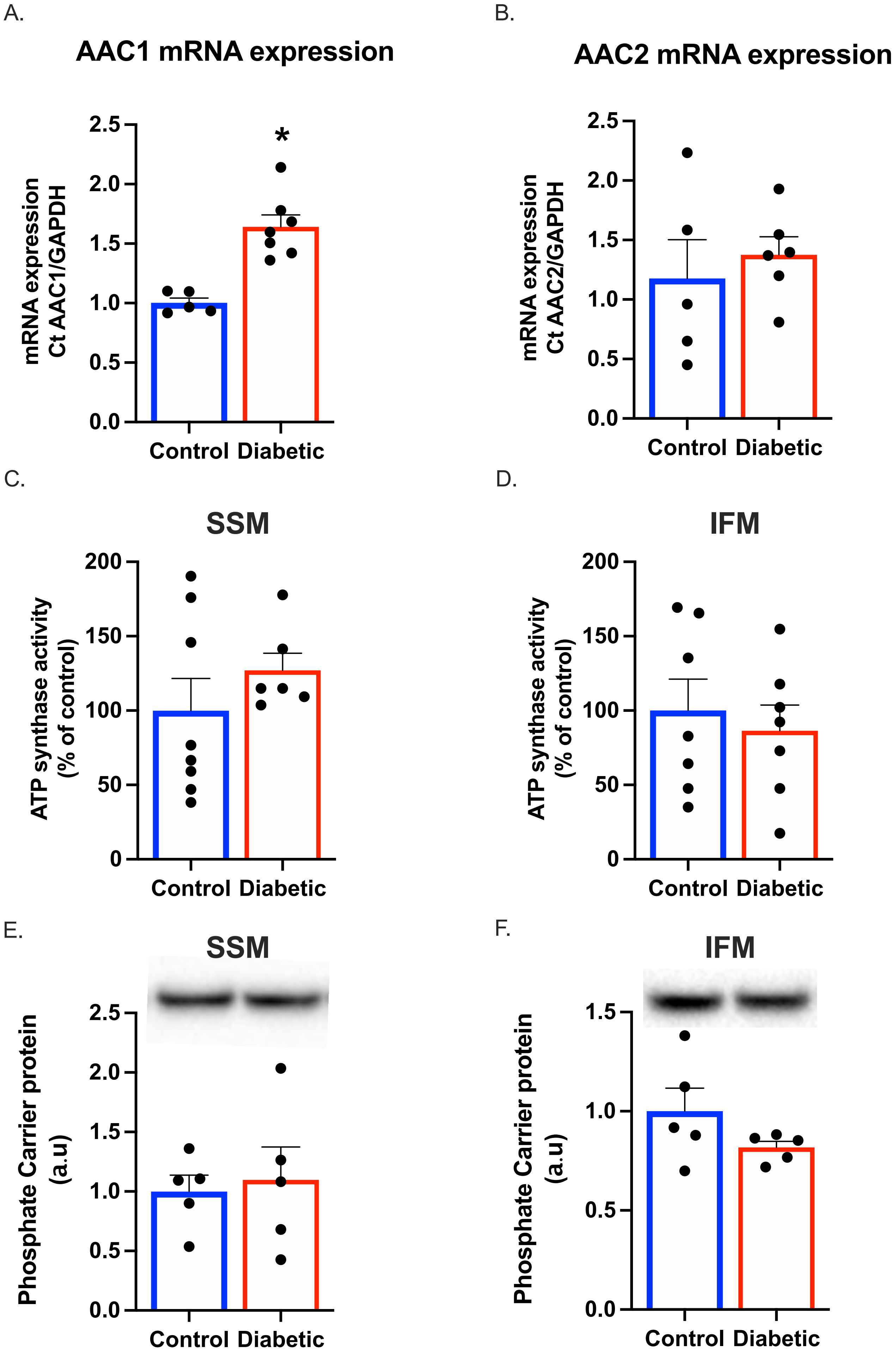

Supplementary Figure Legend. Cardiac expression of AAC1 (A) and AAC2 (B) mRNA in control and diabetic hearts. ATP synthase activity (C-D) and phosphate carrier protein levels (E-F) in SSM and IFM from control and diabetic hearts. * $p < 0.05$ vs. control.
